# Portal Hypertension among Patients with Chronic Liver Disease Admitted to the Department of Internal Medicine of a Tertiary Care Centre

**DOI:** 10.31729/jnma.8294

**Published:** 2023-10-31

**Authors:** Sabina Khadka, Ananda Aryal, Sagun Karki, Pratik Subedi, Kanchan Bogati, Sunil Baniya, Shrekshya Khadka, Kumar Roka, Asha Shahi

**Affiliations:** 1 Nepalese Army Institute of Health Sciences, Sanobharyang, Kathmandu, Nepal; 2 All Nepal Hospital, Samakhusi, Kathmandu, Nepal; 3 Oxford University Clinical Research Unit, Patan Academy of Health Sciences, Lagankhel, Lalitpur, Nepal; 4 Department of Internal Medicine, Shree Birendra Hospital, Chhauni, Kathmandu, Nepal; 5 Naubise Health Post, Dhunibesi, Dhading, Nepal

**Keywords:** *inpatients*, *liver disease*, *portal hypertension*, *prevalence*

## Abstract

**Introduction:**

Portal hypertension is increased pressure within the portal vein. A portal pressure gradient of more than 10 mmHg is defined as "clinically significant portal hypertension" due to manifestations such as splenomegaly, gastroesophageal varices, ascites, hepatorenal syndrome, hepatopulmonary syndrome, hepatic encephalopathy, and spontaneous bacterial peritonitis. The aim of this study was to find out the prevalence of portal hypertension among patients with chronic liver disease admitted to the Department of Internal Medicine of a tertiary care centre.

**Methods:**

A descriptive cross-sectional study was conducted among patients with chronic liver disease in the Department of Internal Medicine of a tertiary care centre from 1 January 2021 to 31 December 2022 after obtaining ethical approval from the Institutional Review Committee. Convenience sampling method was used. The point estimate was calculated at a 95% Confidence Interval.

**Results:**

Among 247 patients with chronic liver disease, the prevalence of portal hypertension was 38 (15.38%) (10.88-19.88, 95% Confidence Interval). A total of 16 (42.11%) patients were in the age group of 51-60 years and males were 36 (94.74%). Ascites as a complication were found in 4 (10.53%).

**Conclusions:**

The prevalence of portal hypertension among chronic liver disease inpatients in a tertiary care centre was found to be lower than other studies done in international settings.

## INTRODUCTION

Portal hypertension is a condition where the pressure within the portal vein increases. A pressure difference of 6 mmHg or more between the portal venous pressure and hepatic veins (or inferior vena cava) is defined as portal hypertension. The causes of portal hypertension are classified as prehepatic like portal vein thrombosis, idiopathic tropical splenomegaly; intra-hepatic such as cirrhosis, alcoholic hepatitis, sarcoidosis, schistosomiasis, congenital hepatic fibrosis and posthepatic like Budd-Chiari syndrome.^1^

The prevalence of portal hypertension among nonalcoholic fatty liver disease patients in a study is 28.2%.^2^ Several studies have been conducted in Nepal in order to study the clinical profile of chronic liver disease (CLD),^3-6^ however, none of these studies have determined the prevalence of portal hypertension among CLD inpatients.

The aim of this study was to find out the prevalence of portal hypertension among patients with chronic liver disease admitted to the Department of Internal Medicine of a tertiary care centre.

## METHODS

This descriptive cross-sectional study was conducted among the patients with chronic liver disease admitted to the Department of Internal Medicine of Shree Birendra Hospital, Chhauni, Kathmandu, Nepal from 1 January 2021 to 31 December 2022 after obtaining ethical approval from the Institutional Review Committee (Reference number: 858). All CLD patients admitted to the medical ward during the study period with complete data were included in the study. CLD patients in the medical intensive care unit, medical high care unit and patients with incomplete data were excluded from the study. A convenience sampling method was used. The sample size was calculated using the following formula:


$$\begin{array}{ll} \text{n} & ={\text{Z}}^{2}\times \frac{\text{p}\times \text{q}}{{\text{e}}^{\text{2}}}\\ & =1.96^{2}\times \frac{0.50\times 0.50}{0.07^{2}}\\ & =196 \end{array}$$

Where,

n = minimum required sample size
Z = 1.96 at 95% Confidence Interval (CI)
p = prevalence taken as 50% for maximum sample size calculation
q = 1-p
e = margin of error, 7%

After adjusting for 10% non-response rate, the calculated sample size was 218. However, we have included 247 patients. In our study, chronic liver disease inpatients were screened for portal hypertension. In patients with ascites, Serum Ascitic Albumin Gradient (SAAG) was calculated and SAAG >1.1 confirmed the diagnosis of portal hypertension.^7^ For UGI bleed cases in the form of hematemesis or melena, the patients were advised to undergo upper GI endoscopy (Oesophago-Gastro-Duodenoscopy, OGD) and USG doppler study of abdomen. OGD showing the presence of esophageal and fundal varices confirmed the presence of portal hypertension.^8^ USG Doppler assisted in the assessment of the risk of variceal bleeding and in the evaluation of the progression of liver disease.^9^ Data was entered into Microsoft Excel 2007 and analysed using IBM SPSS Statistics version 20.0. The point estimate was calculated at a 95% CI.

## RESULTS

Among 247 patients, the prevalence of portal hypertension was 38 (15.38%) (10.88-19.88, 95% CI). The highest frequency of portal hypertension is seen among the age group 51-60 which was 16 (42.11%) (Table 1).

**Table 1:** Age distribution of portal hypertension among CLD inpatients (n = 38).

Age group (years)	n (%)
31-40	3 (7.89)
41-50	8 (21.05)
51-60	16 (42.11)
61-70	7 (18.42)
71-80	4 (10.53)

**Figure 1:**
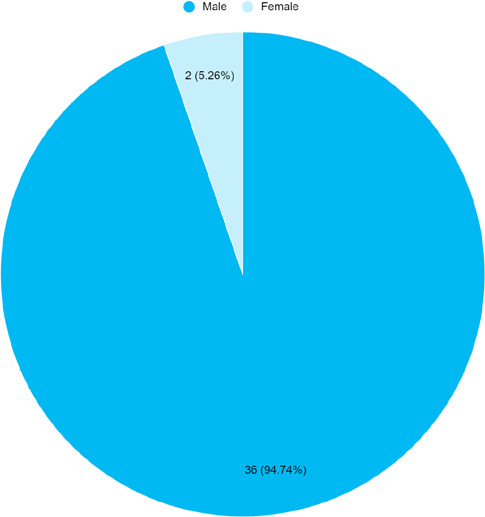
Sex-wise distribution of portal hypertension among CLD inpatients (n= 38).

A total of 36 (94.74%) were males with a male-to-female ratio of 18:1 (Figure 1).

Complications associated were ascites seen in 4 (10.53%) and coagulopathy seen in 2 (5.26%) (Table 2).

**Table 2:** Complications associated with portal hypertension (n= 38).

Complications	n (%)
Ascites	4 (10.53)
Coagulopathy	2 (5.26)
Spontaneous bacterial peritonitis	2 (5.26)
UGI bleed	2 (5.26)
Ascites and coagulopathy	1 (2.63)

## DISCUSSION

The The prevalence of portal hypertension among CLD inpatients was 15.38%. In a similar study, the prevalence of portal hypertension among nonalcoholic fatty liver disease patients was 28.2%.^2^ Similarly, in another international study the portal hypertension prevalence was >90% in all compensated advanced CLD etiologies.^10^ The lower prevalence of portal hypertension in our study than in these studies might be due to differences in the study population. The study population in our study included only military personnel and their families as the hospital provides medical services to these individuals only and the study included inpatients only.

The clinical profile of CLD patients have been studied widely in Nepal.^3-6^ A study done in Nepal determined the signs, symptoms and complications of portal hypertension.^3^ Another study in Nepal aimed to find out the upper GI endoscopic findings of patients presenting with liver cirrhosis with portal hypertension.^4^ Similarly, other studies in similar settings had studied alcoholic liver disease in terms of its presentation, lab findings and complications.^5,6^ Our study is different from these studies in a view that we had studied the prevalence of portal hypertension among CLD inpatients in a tertiary care center for the first time in Nepal. In our study, the majority of the study participants with portal hypertension were middle-aged males. None of the patients with portal hypertension in our study were <30 years old. From this finding, portal hypertension is more common among middle-aged individuals. On the other hand, 94.74% of the patients with portal hypertension in our study were males. Hence, male predominance was observed in our study. In a study done in central Nepal, cirrhosis was seen mostly among middle-aged males^11^ and one of the most important etiology of portal hypertension is cirrhosis.

The complications associated with portal hypertension include ascites, acute variceal bleeding, hepatic encephalopathy, hepatorenal syndrome etc.^12,13^ In our study, the majority of the patients with portal hypertension had associated ascites followed by coagulopathy, spontaneous bacterial peritonitis and upper GI bleeding. A study done in Bhairahawa, Nepal has studied the upper gastrointestinal findings of patients presenting with liver cirrhosis with portal hypertension. In this study Esophageal Varies, important cause of upper GI bleeding were seen in 51 (57.3%) patients,^4^ however in our study UGI bleed was seen in 5.26% of portal hypertension patients.

There are certain limitations associated with this study. As the study is conducted in a tertiary care centre in Nepal, the results may not be generalized to whole population of Nepal. Similarly, the study participants represent this tertiary care centre only because participants belonged to the military background and their families.

## CONCLUSIONS

The prevalence of portal hypertension among patients with CLD admitted to tertiary care centre was found to be lower than other studies done in international settings. Since portal hypertension is one of the important complications of CLD, further studies need to be conducted in Nepal to determine its prevalence.
